# Relating protein functional diversity to cell type number identifies genes that determine dynamic aspects of chromatin organisation as potential contributors to organismal complexity

**DOI:** 10.1371/journal.pone.0185409

**Published:** 2017-09-25

**Authors:** Daniela Lopes Cardoso, Colin Sharpe

**Affiliations:** Institute of Biomolecular and Biomedical Science, School of Biological Sciences, University of Portsmouth, Portsmouth, United Kingdom; Saint Louis University School of Medicine, UNITED STATES

## Abstract

Organismal complexity broadly relates to the number of different cell types within an organism and generally increases across a phylogeny. Whilst gene expression will underpin organismal complexity, it has long been clear that a simple count of gene number is not a sufficient explanation. In this paper, we use open-access information from the Ensembl databases to quantify the functional diversity of human genes that are broadly involved in transcription. Functional diversity is described in terms of the numbers of paralogues, protein isoforms and structural domains for each gene. The change in functional diversity is then calculated for up to nine orthologues from the nematode worm to human and correlated to the change in cell-type number. Those with the highest correlation are subject to gene ontology term enrichment and interaction analyses. We found that a range of genes that encode proteins associated with dynamic changes to chromatin are good candidates to contribute to organismal complexity.

## Introduction

Eukaryotic organisms show increased complexity, when considered across a broad phylogeny, but is it possible to identify specific groups of genes, related in structure or function, that make a major contribution to this feature? In this paper we take a simple, three-step approach to identify such groups. The first step is to quantify the functional diversity of genes for a range of metazoans, the second is to identify those genes whose change in functional diversity correlates positively with a measure for increased complexity across these species. The final step is to look both for enrichment of common features associated with the identified genes, and for interaction networks involving them, as this is likely to indicate cellular processes associated with complexity. One requirement is an appropriate measure of organismal complexity; there are many indicative changes in anatomy and morphology, but these do not lend themselves to quantification, instead, the main reliance in recent years has been on the number of different cell types within an organism [1,2].

As much as it is accepted that organismal complexity increases across the eukaryotic phylogeny, it is also clear that the underlying mechanism will involve changes in the expression of genes that determine the formation and function of differentiated cells. It was realised from an early stage in the genomic era [3], however, that there is insufficient variation in total gene number, from species to species, to account for increased complexity. Instead, two predominant components have been identified: first, an increase in the ability to regulate patterns of gene expression through the use of cis-acting regulatory elements [4,5] and second, changes to the coding capacity of the genes themselves [6]. A complete understanding of the development of organismal complexity requires both components to be considered. The Encode project [7] is working towards a comprehensive analysis of regulatory elements, but there is currently insufficient information on promoters, enhancers and silencers across a wide range of species for regulatory elements to be included in this analysis. It is possible, however, to use the annotation information collected in genomic databases such as Ensembl [8] to consider changes in the protein-coding capacity of genes. Indeed, variables within a genome such as the degree of alternative splicing, which generates protein isoforms, and the number of motifs and domains, that often determine processes such as DNA binding or interactions with other proteins, have both previously been shown to exhibit a strong relationship with organismal complexity [1,2]. In addition, a measure of the proteome size, based on the total number of translated amino acids, which combines gene number with the number and length of all known isoforms, also demonstrates a correlation with organismal complexity, as defined by cell-type number [9]. In summary these studies demonstrated that genome-wide values for these variables correlate with organismal complexity. The aim in this paper, however, is to quantify the change in these variables for specific genes across a range of species and correlate this change with organismal complexity

At the level of an individual gene family, we have previously examined the NCoR family of corepressors across the Deuterostomes [10] and identified changes in three variables that affect the range of proteins produced by this gene family. These are first, an increase in gene number, since there is a single gene in the sea urchin, but two paralogues in vertebrates (NCoR1 and NCoR2). It is often the case that, following duplication, the daughter genes can share existing, or take on new activities (sub- and neo-functionalisation)[11,12]. Second, an increase in isoforms due to alternative splicing and the use of multiple promoters [13–15], and third, an increase in the number of motifs and domains (specifically CoRNR boxes) that determine the specificity of the interaction of NCoR with a wide range of nuclear receptors [16].

In this paper we establish a simple algorithm to quantify the functional diversity of eukaryotic genes based on these three variables. The data is extracted from the Ensembl genome databases for nine species ranging from the nematode worm *C*. *elegans* to humans. Since organismal complexity is likely to involve proteins that determine which genes are expressed in a particular cell type, the analysis assesses over 2000 human genes broadly associated with gene expression, and their annotated orthologues in the other species. Genes that are strongly correlated with cell-type number, as a convenient measure of complexity, are selected for further analysis. The first approach is to use gene ontology to search for descriptive terms that are used more frequently for the set of selected genes than for the set of input genes. Since the motif component contributes to the capacity for interaction with other proteins, the second approach screens for networks of interacting proteins amongst the selected human genes. We find that those genes whose functional diversity correlates with increased complexity are predominantly involved in dynamic aspects of chromatin organisation.

## Results

### Generating a measure of functional diversity, D_F_

The simple algorithm for functional diversity (D_F_) takes into account the number of paralogues, the number of protein isoforms from a single gene and the number of annotated motifs and domains within each protein-coding transcript of the gene. The information was extracted from genome sequences available in the Ensembl database (Release 87)[8] and details of the criteria, genomes and algorithm are provided in the Methods section.

In this paper, we limit the analysis to genes broadly associated with transcription, as listed in the AnimalTFDB 2.0 database of 2087 human genes [17], although the same approach could be used for other subsets, such as proteins involved in signal transduction, or for the entire genome. The list was used to select orthologues from the macaque, mouse, chick, Xenopus, Fugu, Ciona, Drosophila and Caenorhabditis genomes (see Methods for details) and their D_F_ values calculated (lists in S1 and S2 Data).

### Correlating the change in functional diversity to cell-type complexity

To identify genes with a potential role in organismal complexity, candidates were selected whose increase in D_F_ had a strong positive correlation with the change in cell-type number across the chosen phylogeny. Genes were selected that had a significant Pearson’s correlation value (p<0.05) in a two-tailed t-test that takes into account the number of genomes considered (see Table 1).

**Table 1:** Selection of strongly correlating genes. Human genes with orthologues first seen in either *C*. *elegans*, *D*. *melanogaster* or *T*. *rubripes* and then a total of at least six orthologues (providing four degrees of freedom) were processed. Human genes from sets of orthologues that had correlation values greater than a boundary value, set as the correlation value with a probability of p< 0.05 in a two-tailed t-test, were selected. This identified 100 genes first seen in *C*. *elegans*, 50 genes first seen in *D*. *melanogaster* and 48 genes first seen in *T*. *rubripes*.

Genome	Assembly	Orthologues	r value p<0.05	No. genes
*Caenorhabditis elegans*	WBcel235	9	0.66	60
		8	0.71	22
		7	0.75	14
		6	0.81	4
*Drosophila melanogaster*	BDGP6	8	0.71	25
		7	0.75	21
		6	0.81	4
*Takifugu rubripes*	FUGU4.0	6	0.81	48
			Total	198

The initial selection was for human genes with an orthologue in *C*. *elegans* and first all, then six, then five, then four of the remaining species. The process was repeated for human genes that lack an orthologue in *C*.*elegans* but have one in *D*. *melanogaster*, again increasing the degree of correlation for genes that lack an annotated orthologue in up to two of the remaining species. Finally, genes with a significant positive correlation value were chosen from those human genes that have orthologues in each of the vertebrate species (Table 1).

**Fig 1:**
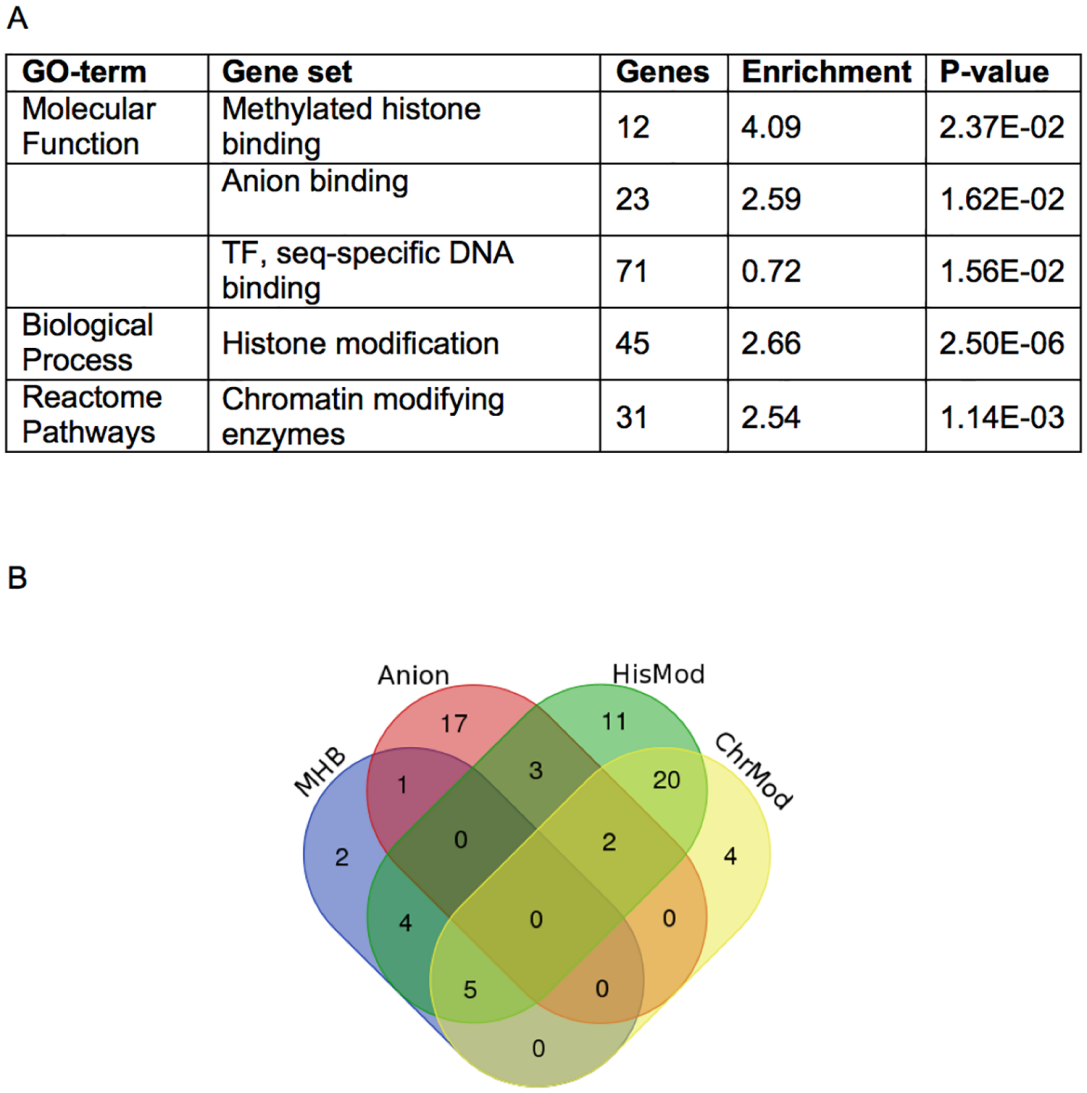
GO-term enrichment analysis. A. Highly correlated genes were subject to GO-term enrichment analysis using AmiGO2 [18] and the Panther Classification system [19,20], using the functions Molecular Function, Biological Process, Reactome Pathway and Cellular Localisation, though the last did not identify any significant gene sets. Significance is determined as 2.5 fold enrichment and a probability of p<0.05. DNA sequence-specific transcription factors have an enrichment of 0.72 indicating that they are under-represented. B. The four enriched gene sets include 112 genes, which in a Venn diagram identifies 69 independent genes. (MHB, Methylated histone binding; Anion, Anion binding; HisMod, Histone modification; ChrMod, Chromatin modifying enzymes).

The 198 significant genes (9.6% of input) (complete list in S3 Data) were then pooled and analysed using AmiGO2 v2.5.5 [18] for significant enrichment in the Panther GO-terms complete analysis [19,20] for Molecular Function, Biological Process and Cellular Component and, in addition, for genes enriched for the Reactome pathways term, in each case comparing to the reference set of 2087 genes from the AnimalTFDB 2.0 database [17]. Sets were selected that demonstrated more than a 2.5 fold enrichment with a significant probability of p<0.05. Whilst individual genes may contribute to organismal complexity, finding aspects held in common between the significantly correlating genes may additionally point towards processes that underpin complexity. The cellular component enrichment analysis did not return a significant set (Fig 1A and 1B and complete tables from the GO-term complete analysis in S4 Data).

**Fig 2:**
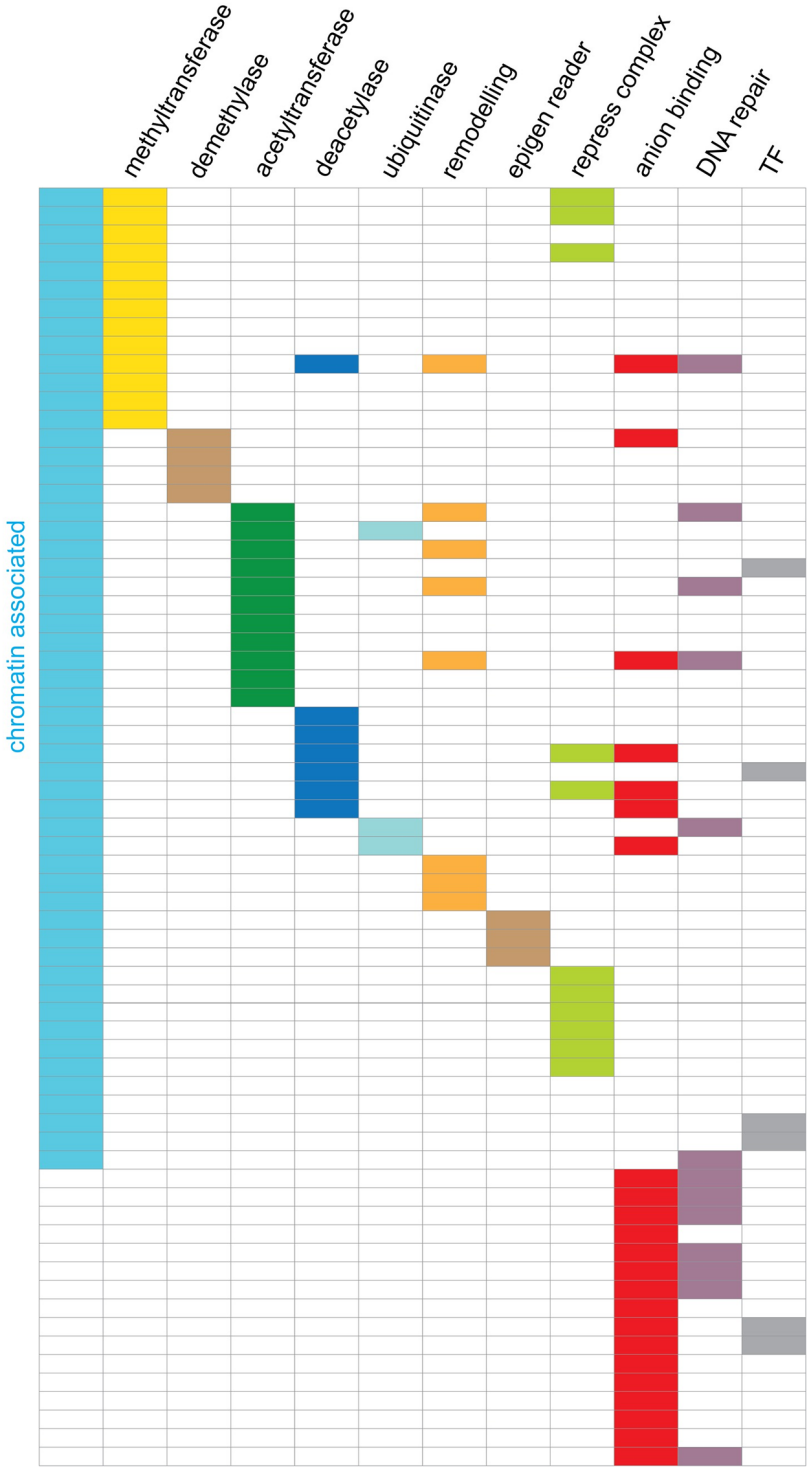
GO-term enrichment identifies sets of genes primarily involved in dynamic chromatin structure and function. Details from the GeneCards database were used to further sort the sets into specific functions shown by the columns. Each row is a gene and their identity is listed in the Supplementary information (S6 Data) the 53 genes associated with chromatin function are depicted by the blue column. The genes cover a wide range of chromatin-associated functions. The two blank rows represent MED24 and TBL1Y which are in the GO-term ‘histone modification’ but do not contribute to the functions in the columns. epigen reader, epigenetic readers; repress complex, chromatin repressive complexes;,TF, DNA sequence specific transcription factors.

After accounting for redundancy between the four enriched sets, 69 genes were identified (Fig 1B and S5 Data) of which 52 (75.3%) were associated with the three sets directly relating to histone and chromatin modification. For the Molecular Function term ‘Anion binding’ 7 out of 23 genes were also present within the extended chromatin group (Fig 2).

The selected genes were analysed in finer detail using functional descriptions from within GeneCards (www.genecards.org). This identified 53 genes (77%) directly involved in dynamic chromatin organisation, since this approach additionally identified PHF12 as a component of the Sin3A, histone deacetylase complex [21] (Fig 2). The subsets included histone methylases and demethylases and histone acetyltransferases and deacetylases, which are involved in the covalent modification of histones associated with both activating and repressing gene expression [22,23]. In addition, subsets identified components of remodelling complexes, such as SWI/SNF [24], and repressive chromatin complexes including the polycomb repressor complexes [25], but no one group predominated. At least 5 genes that have functions associated with the dynamic organisation of chromatin also have a role in DNA repair such as the components of the NuA4 HAT complex that also plays a role in nucleosome remodelling and DNA repair [26,27]. In addition, there are 9 genes within the Anion binding set involved in various forms of DNA repair. In contrast to the proteins associated with the dynamic organisation of chromatin, typical transcription factors represented by the GO term Molecular Function ‘transcription factor activity, sequence-specific DNA binding’ were under-represented, appearing as a depleted component in the Molecular function term (Fig 1A).

### Identifying a network of interacting genes

**Fig 3:**
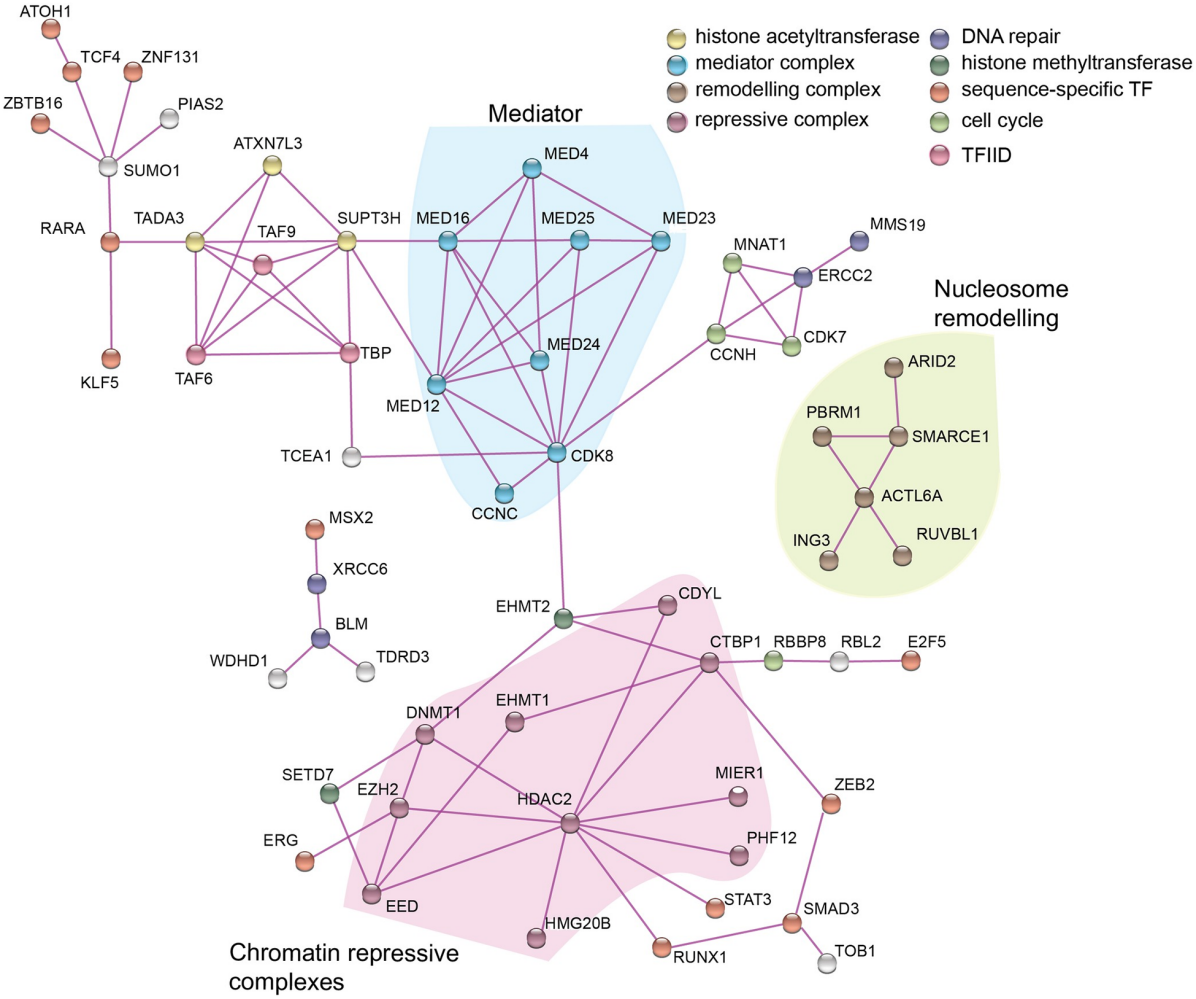
Interacting networks of the highly correlating proteins. The 198 highly correlated human genes were entered into STRING and interactions confirmed by direct experimental evidence with a high confidence level (0.700) selected. Networks of more than three components were selected and the output recoloured in Adobe Illustrator 2014 to highlight protein functions shown in the key. There is no significance to the length or direction of the connections. Three clusters of related function, Mediator, nucleosome remodelling and chromatin repressive complex are grouped in shaded areas.

One mechanism underpinning the contribution of protein functional diversity to organismal complexity is likely to be an increased ability to interact with other proteins. Interactions may drive complexity by expanding the number of component proteins within a complex or by increasing the complement of proteins that can contribute to a complex, as seen for polycomb repressor complex 1, which, in humans selects one from five paralogues (CBX2, 4, 6, 7 and 8)[28,29]. Identifying the interactions between the 198 human genes that are highly correlated with cell-type number may, in addition to GO-term analysis, indicate processes that contribute to organismal complexity. To do this, the gene list was entered into the String program [30] set solely to consider experimental data for interaction at a high level of confidence (Fig 3).

Sixty of the selected genes (30% of input) segregated into three interaction networks of more than 3 components, one of which contained 44 genes. Of the 60 genes, 28 were previously identified by GO-term enrichment, so together the two approaches identified 98 genes (49% of the highly correlated genes). The most connected gene is the histone deacetylase, HDAC2 (10 connections), a component, along with its paralogue, HDAC1, in the NuRD, CoREST and Sin3 repressive complexes [31]. Following that are CDK8 (8 connections) and MED12 (7 connections), which are both part of the Mediator complex that forms a physical link between transcription factors at distal enhancers and the basal transcription machinery at the proximal promoter [32].

The interaction map identified at least three coherent clusters (Fig 3). The first, centered on HDAC2, consists of proteins that contribute to chromatin repressive complexes and in addition has links to the histone methyltransferases, SETD7 and EHMT2, consistent with the enrichment in histone methyltransferases seen in the GO-term analysis. EHMT2, known as G9A, can also interact with the PRC2 complex that contains EED and EZH2 that are also members of this cluster [33,34]. The identification of components of repressive chromatin complexes highlights that gene repression is likely to be as important as the activation of gene expression. This is specifically the case in the maintenance of the embryonic stem cell pluripotency in vertebrates that involves both PRC1 and PRC2 [35,36]. It is worth noting though that the GO-term analysis identified a range of chromatin modifying proteins that included both repressive components and activating components such as the histone acetyl transferases and three genes of this class are also present in the interaction analysis (Fig 3).

The second cluster is a freestanding set of six genes associated with chromatin remodelling that are predominantly components of the SWI/SNF complex [24]. This is again consistent with the findings from the GO-term enrichment analysis. The third cluster consists of 8 proteins that contribute to the Mediator complex [32], a connection that was not apparent from the GO-term analysis. In addition there is a small cluster that links three components of the TFIID transcription complex to three components of the SAGA histone acetyl transferase complex, both involved in the recruitment of TBP to the proximal promoter [37].

## Discussion

Protein coding aspects of the genome that correlate with organismal complexity increase the information content of the genome through proteome expansion, driven by alternative splicing [2], and the addition of protein domain families [1,9]. In this paper we use a simple algorithm, based on the increase in the number of paralogues, isoforms and protein domains to quantify the functional diversity of genes encoding transcription-associated proteins. Genes are then selected across nine model organisms, based on the correlation of functional diversity with organismal complexity. Finding enrichment for GO-terms and highlighting groups of interacting proteins identifies sets of genes involved in dynamic processes affecting chromatin, particularly epigenetic modification, nucleosome remodelling, DNA repair and the ability to link distal enhancers to proximal promoters through the Mediator complex. Sequence-specific, DNA-binding transcription factors are notably under-represented. These, however, are not general properties of these classes of proteins, as the average D_F_ values, for the GO terms ‘nucleic acid binding transcription factor activity’ and ‘chromatin binding’ show a similar trend across the phylogeny and there is no significant difference between these terms compared to the input data when either the worm or human data sets are considered (Supplementary information, S7 data).

For simplicity, the protocol uses data from nine annotated genomes within the Ensembl site, representing many of the major model organisms. Although additional genomes are available, few are currently annotated to the required depth or accuracy to be used in this approach. Of the algorithm components, the value for the number of paralogues is likely to be the most accurate. The quantification of isoforms, however, depends on the experimental identification and annotation of transcriptional start sites and alternative splicing, which has been extensively surveyed for human genes [7], but currently less so for other species. Similarly, the domain count depends on the accuracy and completeness of the Prosite profiles database [38]. A shortfall in the genome annotation data will cause an underestimate in the calculated functional diversity of proteins in that species, which in turn may affect the correlation with organismal complexity. The simplicity of the pipeline, however, means the output can both be updated, as revised versions of each genome appear on the Ensembl website, and extended as additional genomes are annotated to sufficient depth.

Having calculated a value for the functional diversity (D_F_) of each gene, the next step relates changes in this value to changes in organismal complexity. The primary criterion is a strong positive correlation between the change in D_F_ of a gene and the change in cell-type number, widely used as a measure of organismal complexity [1,2]. Since, for our purpose, the absolute value of the cell type number is less important than the ratio of cell-type numbers across the species, we believe this measure currently provides the most practical and reasonable estimate for the change in organismal complexity. The approach then used GO-term enrichment and experimentally documented physical interaction as filters to highlight sets of human genes with common features. Whilst we do not discount the part played by individual genes that fall outside these sets, the sets indicate groups of genes that contribute to common processes. It is then our hypothesis that the common processes are good candidates to influence organismal complexity.

The focus on changes to protein coding capacity, as a measure of functional diversity, is a constraint, since the contribution of changes in cis-acting regulatory elements (CAREs), has not been considered. It has been suggested that cis-elements underpin many of the changes seen between species [4]; as novel enhancers arise, they drive new patterns of gene expression, without compromising the existing functions of the gene. A well-documented example is the formation of pelvic spines in response to the transcription factor, Pitx1, binding to a specific enhancer in the genome of marine, but not freshwater, populations of sticklebacks [39]. In contrast, changes to the coding sequence of a protein are considered more likely to disrupt the existing activity of the protein, rather than to provide additional functions [4]. Despite rapid advances in the Encode project [7], only a fraction of CAREs within the human genome have been annotated, and even fewer in other species. The lack of data currently excluded the use of this component of functional diversity in this study.

The route by which CAREs contribute to functional diversity and organismal complexity differs substantially in character from that contributed by coding capacity-dependent functional diversity. Increased CARE diversity introduces the potential for novel patterns of gene expression, mediated by the binding of existing, sequence-specific transcription factors. In essence, the transcription factor proteins do not need to change and instead, diversity depends on a change in the number of CAREs. This is consistent with the fewer than expected transcription factors identified in the two approaches taken here. In contrast, the three mechanisms underlying the functional diversity described in this paper involve increases in the proteome through the formation of paralogues, the generation of isoforms or through the acquisition of protein domains and motifs. The relative contribution that changes to CAREs and to the functional diversity of proteins each make either to the initiation of new cell types or to providing the capacity for novel cell-type activities, however, cannot be determined at this stage.

**Fig 4:**
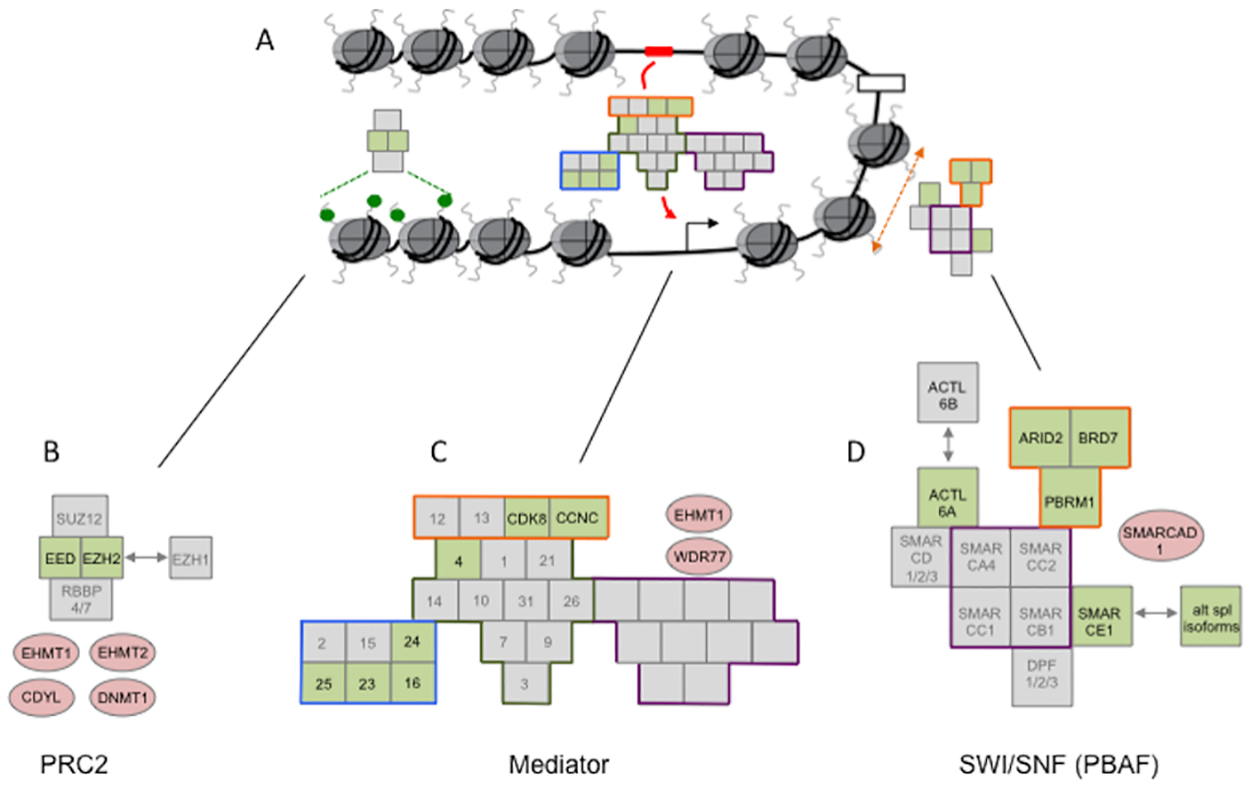
Three representative multiprotein complexes illustrate the mechanisms that underpin complexity. A. Diagrammatic representation of chromatin illustrating the repressive effects of PRC2 via the methylation of histone tails (green dots), the function of Mediator in linking distant enhancers (red box) with proximal promoters (black arrow) and the role of SWI/SNF (PBAF) remodelling complex in the rearrangement of nucleosomes. B. PRC2 consists of four core components of which two, EED and EZH2, feature in the list of selected proteins (shaded green). Additional diversity is generated by the exchange of paralogues EZH2 and EZH1. PRC2 also interacts with a range of proteins (pink ovals) identified in the screen. C. Mediator complex consists of around 30 components divided into head (purple outline), middle (green outline), CDK8 module (orange outline) and tail (blue outline). Numbers in the boxes refer to the MED protein nomenclature, 25 = MED25. Proteins from genes whose functional diversity correlates with organismal complexity are shaded in green and include four of the six tail domain components. D. The SWI/SNF, PBAF complex includes five proteins encoded by genes selected in this screen (shaded green). All three of the PBAF specific genes are included (orange outline). ACTL6A can provide additional diversity by exchanging with its paralogue ACTL5B, whilst SMARCE1 (BAF57) exists in a number of alternatively spliced isoforms, some of which are specifically expressed in neurons.

The three mechanisms underlying functional diversity can be illustrated by reference to three protein complexes that contain components identified in this paper (Fig 4). For example the Drosophila polycomb complex component, E(z), has two paralogues in humans, EZH1 and EZH2, either of which can contribute to PRC2, increasing the diversity of this complex [25,40].

The Mediator complex contains over 20 component subunits and whilst many are conserved from *C*. *elegans* to humans, at least 8 are found only in the higher vertebrates [41]. There are mammalian paralogues of subunits of the kinase module of Mediator that are likely to expand the range of functions of this module, whilst tail module components found only in higher vertebrates such as MED25, identified in the interaction analysis, provide a specific capacity for the interaction with nuclear receptors [32,42]. Indeed, four out of six of the tail components were identified in the screen (MED16, MED23, MED24 and MED25) and MED23 has also been shown to interact with components of the splicing machinery such as hnRNP L to modulate alternative splicing [43]

SWI/SNF in humans differs from that seen in *C*. *elegans* and *D*. *melanogater* by having the option to use different subunits. These notably include two distinct ATPases, BRG1 (SMARCA4) and hBRM (SMARCA2) that define the PBAF and BAF remodelling complexes and the three units that are specific to PBAF (ARID2, BRD7 and PBRM1) were identified in the screen. In addition, ACTL6A (BAF53a) is interchangeable with its paralogue ACTL6B (BAF53b), the different subunit compositions giving a diverse range of remodelling complexes [24] that can also be restricted to specific stages in the differentiation of a cell [44]. Furthermore, all of the genes encoding components of the SWI/SNF remodelling complex identified in the first GO-term screen encode between 2 and 8 GENCODE basic isoforms with the highest transcript support levels (TSL1 and 2) in humans. Little is currently known about the function of these isoforms, though several isoforms of SMARCE1 (BAF57) are neuronal specific in mammals [45]. The five isoforms of PBRM1 differ in the number or type of domains present in the protein, which is thought to alter the way that the protein interacts with acetylated nucleosomes [46]. Given the major contribution of isoforms to our measure of diversity it would be interesting to explore the functions of more of these isoforms. We have only considered the SWI/SNF components, but it is clear that protein isoforms contribute to the diversity of most, if not all, of the proteins identified in these screens.

In a previous paper we noted that the addition of CoRNR box motifs, responsible for the interactions with nuclear receptors, contributed to the increased complexity of the corepressor NCoR2 [10]. This is not uncommon and was verified in over 25% (12/44) of the chromatin-associated genes identified in this study. Examples include the addition of a single new domain, such as the SCA7 (IPR013243) domain in human ATXN7L3 (NP_064603) that is not seen in the Drosophila orthologue, Sgf11, or the presence of 7 repeated ankyrin domains (IPR002110) seen in human EHMT1 (NP_079033), but not in the nematode set-11 gene. In some cases, it is not the addition of a new domain, but the expansion of an existing domain that occurs and examples of this include WD repeats in human EED (NP_003788, 6 repeats), a component of PRC2, compared to the nematode mes-6 (4 repeats) and PHD-type Zn fingers (IPR001965) in human NSD1 (NP_071900, 5 repeats), compared to Drosophila Mes-4 (NP_733239, 3 repeats).

The increased D_F_ value of the selected genes suggests a mechanism by which the number of interactions can be increased to fulfil the requirements of greater complexity. In addition there is evidence that some of the selected genes may contribute to the formation of different cell types. One route might be through the modification of stem cell activity in response to a greater functional diversity of the proteins that modulate epigenetic status to either maintain or promote differentiation [35,36]. For example, EZH2 and EED, are histone methyltransferases within the PRC2 complex that generate di- and tri-methyl marks on H3K27 that form repressive chromatin [47] and loss of function Eed mutant embryonic stem cells express markers of neuronal differentiation [48]. Gene editing to manipulate isoform production or to delete specific domains, however, is likely to be more informative of the roles of these genes in determining the complexity of organisms since it is the increase in D_F_ value, rather than the presence or absence of the gene that correlates with complexity.

In conclusion, we have used a simple approach to identify candidate genes whose encoded proteins may underpin organismal complexity by extracting the data for paralogues, isoforms and domains from the Ensembl genome databases for 9 multicellular animals. Orthologue sets with a strong positive correlation to cell-type number, as an accessible measure of complexity, were then subject to GO-term and interaction analysis to identify common features and processes. DNA sequence-specific transcription factors are notably under-represented in the selection, which is enriched for proteins involved in dynamic interactions of the chromatin. This makes a clear distinction between complexity driven by transcription factors binding to an increasingly diverse array of enhancer elements, which requires little change in the proteins, and complexity driven by non-sequence-specific events at the level of chromatin structure and function that often involve a toolbox of protein complexes with increasingly diverse components [49]. Whilst the increasing range of components within multi-subunit complexes that regulate the dynamic structure of chromatin have been widely discussed [25,29,41,49], we believe this is the first analysis to link organismal complexity to diverse chromatin processes based simply on objective criteria.

## Methods of analysis

### Data collection

First, an input file of human transcription associated genes was derived from the AnimalTFDB 2.0 database (http://bioinfo.life.hust.edu.cn/AnimalTFDB/) [17], which uses the TFs prediction pipeline from Pfam [50] to identify 2308 genes. Comparison with the Ensembl database resolved this into 2087 distinct human genes that could be indexed with Ensembl gene identifiers, which were then used in this analysis. Orthologues of these genes in *Caenorhabditis elegans* (assembly WBcel235), *Drosophila melanogaster* (BDGP6), *Ciona intestinalis* (KH (GCA_000224145.1)), *Takifugu rubripes* (FUGU 4.0), *Xenopus tropicalis* (JGI 4.2), *Gallus gallus* (5.0), *Mus musculus* (GRCm38.p5) and *Macaca mulatta* (Mmul_8.0.1) were then identified using a Python script to access the Ensembl Database Release 87 via REST APIs. The script is available at (https://github.com/GenDataPro/GenDataPro)

### Complexity scoring system

The main algorithm was developed in Python v. 3.4 (see: https://github.com/GenDataPro/GenDataPro) and again accesses the Ensembl databases of each of the above species to collect the numbers of paralogues (P) for each of the genes in the input file, selecting the ‘within species paralogue’ criterion. The number of isoforms (I) is a measure of the abundance of protein isoforms, for each gene, generated by alternative splicing and the use of multiple promoters. This is likely to be the least accurate of the values obtained as it depends largely on the annotation of experimentally derived data either from analysis of individual genes or from RNAseq data. Often the number of transcripts includes several that do not encode protein and as a consequence we restricted the analysis to annotated transcripts that are flagged within the database as ‘protein coding’ transcripts. The number of motifs (M), is based on the parts of the protein that are involved in a specific activity with a defined outcome, such as protein or DNA interaction domains. This value is the sum of all the motifs for each isoform within the gene. This information is collected from Ensembl Prosite Profiles data [38]. Whilst many domains are predictable by sequence comparison, this figure is still likely to be an underestimate as short motifs, such as the short linear motifs of NCoR corepressors are not included [51,52].

### Cleaning and formatting the data

On the Ensembl databases, orthologues do not always share the same official gene name. To simplify the interpretation, an extra field was generated to group orthologues of a gene, indexed by the official Human gene symbol. This means that a given orthologous gene of a Human gene will have two fields designated for gene name, its own and the Human gene name reference. For certain genes (particularly zinc finger-containing transcription factors) the large number of domain repeats within some genes and the large number of paralogues identified these as outliers. Consequently, we transformed all values as a logarithm to the base 2, which maintained these genes within the analysis but removed the bias of outlying genes. The transformed D_F_ value is therefore:

$$D_{F}=log_{2}\text{P}+log_{2}\text{I}+\;\sum_{I=1}^{I=n}log_{2}M$$

For the first correlation analysis, orthologues from C. elegans to humans, used human genes with a ‘one-to one’ orthologue in at least four of the remaining seven species. The same approach was taken for genes first seen in Drosophila and genes first seen in Takifugu (see Table 1). From the initial 2087 human genes this identified 198 qualifying human genes whose orthologue set had a positive correlation with cell-type number that was statistically significant to less than p = 0.05 in a two-tailed t-test taking into account the degrees of freedom available from the number of orthologues in the gene set. Cell-type number was based on data in Vogel and Chothia [1,53] taking *Caenorhabditis elegans* as having 29 different cell types, *Drosophila melanogaster*, 60, *Ciona intestinalis*, 71, *Takifugu rubripes*, 114, *Xenopus tropicalis*, 121, *Gallus gallus*, 150, *Mus musculus*, 157 and *Macaca mulatta* and *Homo sapiens* as 171. The Pearson’s correlation coefficient between cell type number and the D_F_ values was determined within the Excel spreadsheet.

### Gene ontology and interaction analysis

The 198 qualifying human genes were analysed using AmiGO2 v2.5.5[18] for significant enrichment in the GO complete terms, Molecular Function and Biological Process and Cellular Component. In addition, we selected for genes enriched within the Reactome Panther classification system [19,20]. In each case we selected sets that showed a greater than 2.5 fold enrichment and a probability of less than p = 0.05 or that were depleted. The set of non-redundant, positively correlating genes was further classified by analysis of basic function using terms within the GeneCards application (www.genecards.org). To identify interaction networks, the 198 qualifying human genes genes were analysed using STRING v10 [30] using solely experimental data as the criteria for interaction at the high confidence level (0.700). Networks of three or more genes were downloaded as interactive svg files and adapted in Adobe Illustrator.

## Supporting information

S1 Data Excel file of original data for all genes in the analysis. (XLSX)

S2 Data Excel file of functional diversity data for all genes and correlation scores across the phylogeny. (XLSX)

S3 Data Excel file of the 198 significantly correlated genes. (XLSX)

S4 Data Original GO-complete data tables. (XLSX)

S5 Data Table of the selected genes by GO-term. (DOCX)

S6 Data Table of the selected genes by GenCard function. (DOCX)

S7 Data GO term variation across the phylogeny. (DOCX)
