# Supplementary material for: Relating protein functional diversity to cell type number identifies genes that determine dynamic aspects of chromatin organisation as potential contributors to organismal complexity
Source: PLoS One. 2017 Sep 25;12(9):e0185409. doi: 10.1371/journal.pone.0185409 (PMC5612723; doi:10.1371/journal.pone.0185409)
Supplement: S5 Data — (DOCX) [file pone.0185409.s005.docx]

Supplementary for Fig 1. S5

| **Molecular function** |  | **Biological process** |  | **Reactome** |
| --- | --- | --- | --- | --- |
| *MHB* | *Anion* | *Histone*  *modifications* |  | *ChrMod* |
| CBX8 | AK6 | ACTL6A | RUVBL1 | ACTL6A |
| CDYL | BLM | ATXN7L3 | SETD3 | ARID2 |
| ING2 | CDK7 | BCOR | SETD7 | ATXN7L3 |
| ING3 | CDK8 | BRD8 | SETDB1 | BRD8 |
| KAT8 | ERCC2 | CBX8 | SIRT2 | EED |
| LOXL2 | ILF2 | CDYL | SMARCAD1 | EHMT1 |
| MSL3 | ING2 | CTBP1 | SUPT3H | EHMT2 |
| PHF8 | KDM1B | EED | SUV39H2 | EZH2 |
| TDRD3 | PHF12 | EHMT1 | TADA3 | HDAC2 |
| WDR5 | PMS1 | EHMT2 | TBL1XR1 | HDAC8 |
| ZMYND11 | PSMC3 | EYA3 | TBL1Y | HMG20B |
| ZMYND8 | RARA | EZH2 | UBE2B | ING3 |
|  | RUNX1 | HDAC2 | WDR5 | KAT7 |
|  | RUVBL1 | HDAC8 | ZMYND8 | KAT8 |
|  | SF1 | HMG20B |  | KDM1B |
|  | SIRT2 | HMG2A |  | KDM2B |
|  | SMARCAD1 | HNF1A |  | MSL3 |
|  | TRIB3 | HSF4 |  | NSD1 |
|  | TULP1 | ING3 |  | PBRM1 |
|  | TULP3 | KAT7 |  | PHF8 |
|  | UBE2B | KAT8 |  | RUVBL1 |
|  | UBE2L3 | KDM1B |  | SETD3 |
|  | XRCC6 | KDM2B |  | SETD7 |
|  |  | LOXL2 |  | SETDB1 |
|  |  | MED24 |  | SMARCE1 |
|  |  | MIER1 |  | SUPT3H |
|  |  | MSL3 |  | SUV39H2 |
|  |  | NSD1 |  | TADA3 |
|  |  | PHF8 |  | TBL1XR1 |
|  |  | PRMT2 |  | WDR5 |
|  |  | RBM14 |  | WDR77 |
