# Supplementary material for: Relating protein functional diversity to cell type number identifies genes that determine dynamic aspects of chromatin organisation as potential contributors to organismal complexity
Source: PLoS One. 2017 Sep 25;12(9):e0185409. doi: 10.1371/journal.pone.0185409 (PMC5612723; doi:10.1371/journal.pone.0185409)
Supplement: S6 Data — (DOCX) [file pone.0185409.s006.docx]

Supplementary for Fig 2 S6

| Methyltransferase | Demethylase | Acetylase | Deacetylase | Ubiquitinase | Nucleosome  remodelling | Epigenetic  reader | Repressive  complex | Anion  binding | DNA  repair | Transcription  factor |
| --- | --- | --- | --- | --- | --- | --- | --- | --- | --- | --- |
| EED | KDM1B | ACTL6A | HDAC2 | EYA3 | SMARCAD1 | ZMYND8 | EED | TULP1 | SMARCAD1 | RARA |
| EHMT1 | KDM2B | ATXN7L3 | HDAC8 | UBE2B | ACTL6A | TDRD3 | EHMT1 | TULP3 | ACTL6A | RUNX1 |
| EHMT2 | PHF8 | BRD8 | ING2 | ATXN7L3 | BRD8 | ZMYND11 | EZH2 | SMARCAD1 | ING3 | HNF1A |
| EZH2 | LOXL2 | ING3 | SIRT2 |  | ING3 |  | ING2 | KDM1B | RUVBL1 | MIER1 |
| NSD1 |  | KAT7 | SMARCAD1 |  | RUVBL1 |  | HMG20B | RUVBL1 | EYA3 | HMG2A |
| SETD3 |  | KAT8 | MIER1 |  | ARID2 |  | TBL1XR1 | UBE2L3 | AK6 | HSF4 |
| SETD7 |  | MSL3 | PHF12 |  | PBRM1 |  | BCOR | ING2 | BLM |  |
| SETDB1 |  | RUVBL1 |  |  | SMARCE1 |  | CTBP1 | SIRT2 | ERCC2 |  |
| SUV39H2 |  | SUPT3H |  |  |  |  | CBX8 | UBE2B | ILF2 |  |
| WDR5 |  | TADA3 |  |  |  |  | CDYL | TRIB3 | PMS1 |  |
| WDR77 |  | HNF1A |  |  |  |  | PHF12 | PHF12 | XRCC6 |  |
| PRMT2 |  |  |  |  |  |  |  | AK6 | CDK7 |  |
| SMARCAD1 |  |  |  |  |  |  |  | BLM | RBM14 |  |
|  |  |  |  |  |  |  |  | ERCC2 |  |  |
|  |  |  |  |  |  |  |  | ILF2 |  |  |
|  |  |  |  |  |  |  |  | PMS1 |  |  |
|  |  |  |  |  |  |  |  | XRCC6 |  |  |
|  |  |  |  |  |  |  |  | CDK7 |  |  |
|  |  |  |  |  |  |  |  | CDK8 |  |  |
|  |  |  |  |  |  |  |  | PSMC3 |  |  |
|  |  |  |  |  |  |  |  | RARA |  |  |
|  |  |  |  |  |  |  |  | RUNX1 |  |  |
|  |  |  |  |  |  |  |  | SF1 |  |  |
