# Supplementary material for: Relating protein functional diversity to cell type number identifies genes that determine dynamic aspects of chromatin organisation as potential contributors to organismal complexity
Source: PLoS One. 2017 Sep 25;12(9):e0185409. doi: 10.1371/journal.pone.0185409 (PMC5612723; doi:10.1371/journal.pone.0185409)
Supplement: S7 Data — (DOCX) [file pone.0185409.s007.docx]

Supplementary information, S7 Data


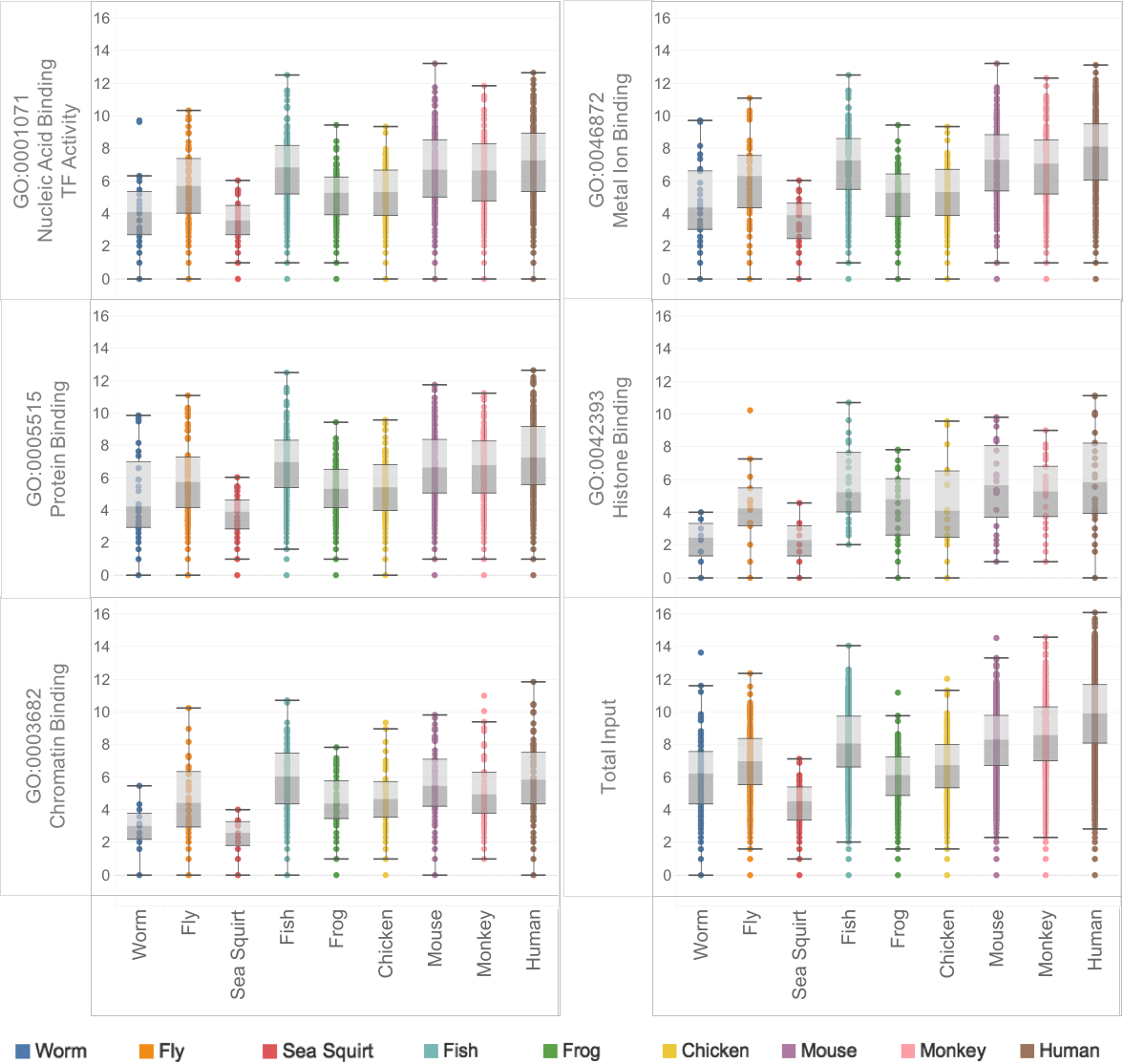


A

B

Human

Worm


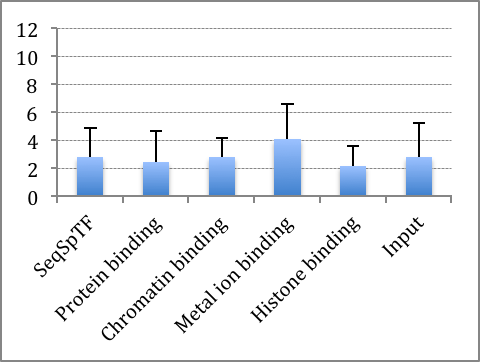

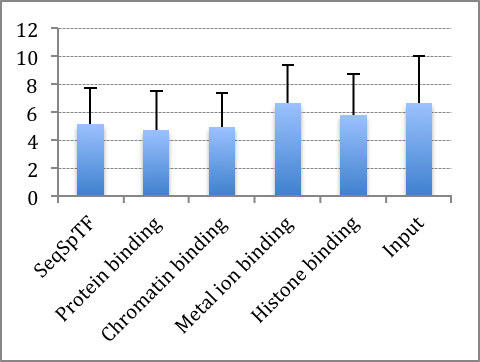


A. Box and whisker plot of D_F_ values for a range of GO-terms across the nine species examined. For each term there is a gradual increase in D_F_ across the phylogeny.

B. Comparing the mean values for D_F_ for a range of GO-terms in either C.elegans (worm) or humans shows in the worm that there is no statistically significant difference between most GO-term gene sets and the input set except for metal ion binding. In the human case three terms are significantly lower than the input (two tailed Student’s t-test) and these include both the nucleic acid TF binding activity (SeqSpTF) and chromatin binding indicating that there is no evidence for selection for increased DF in genes associated with chromatin binding compared to DNA sequence specific transcription factors.
